# Supplementary figures and images for: Mitogenome of the stink bug Aelia fieberi (Hemiptera: Pentatomidae) and a comparative genomic analysis between phytophagous and predatory members of Pentatomidae
Source: PLoS One. 2023 Oct 11;18(10):e0292738. doi: 10.1371/journal.pone.0292738 (PMC10566676; doi:10.1371/journal.pone.0292738)

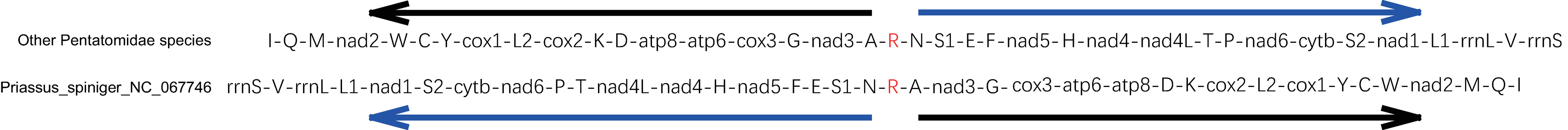

Supplement: S1 Fig — (TIF) [file pone.0292738.s001.tif]

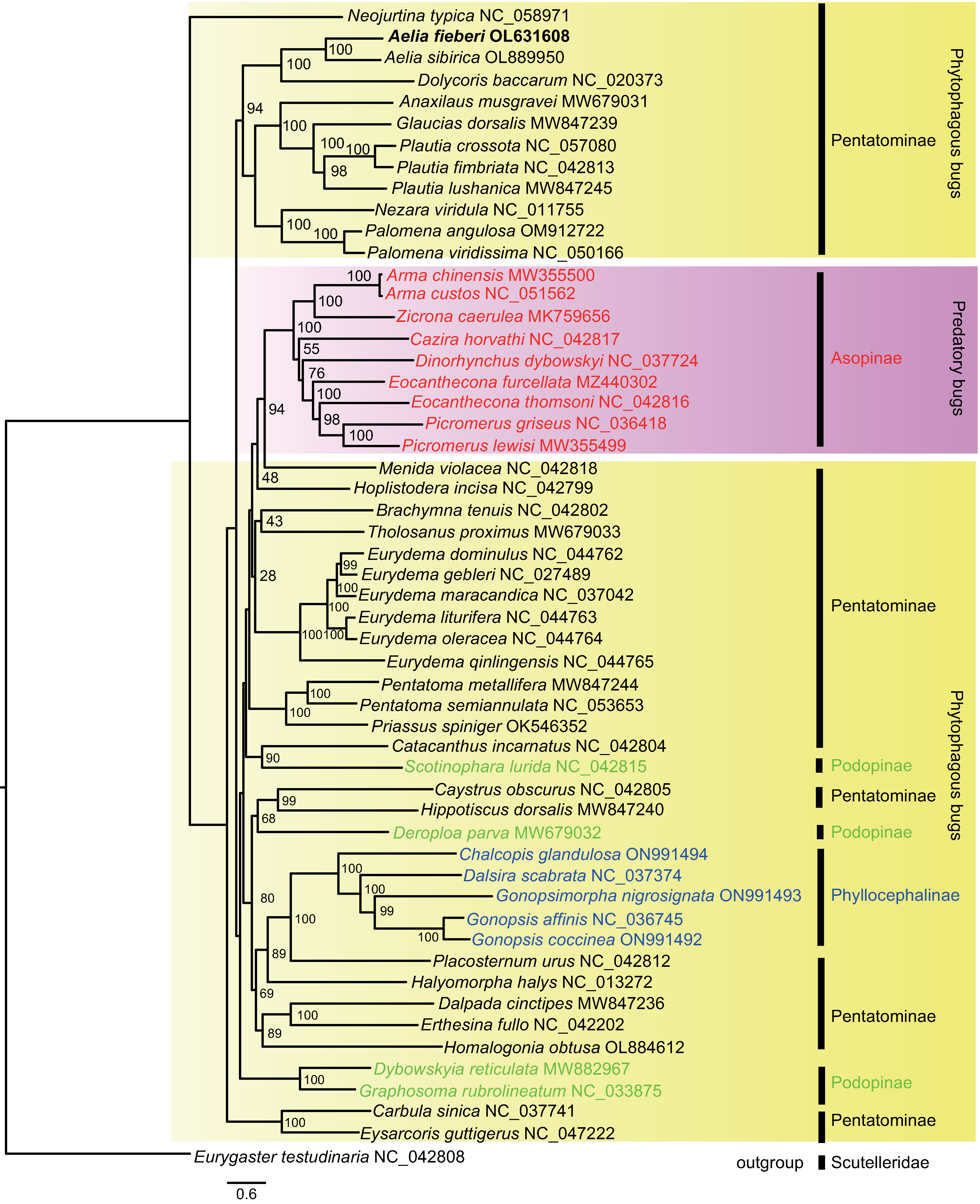

Supplement: S2 Fig — Eurygaster testudinaria (Hemiptera: Scutelleridae) was selected as representative of the outgroup. The bootstrap values were labeled at each node. GenBank accession numbers of sequences were listed after the species name. (TIF) [file pone.0292738.s002.tif]

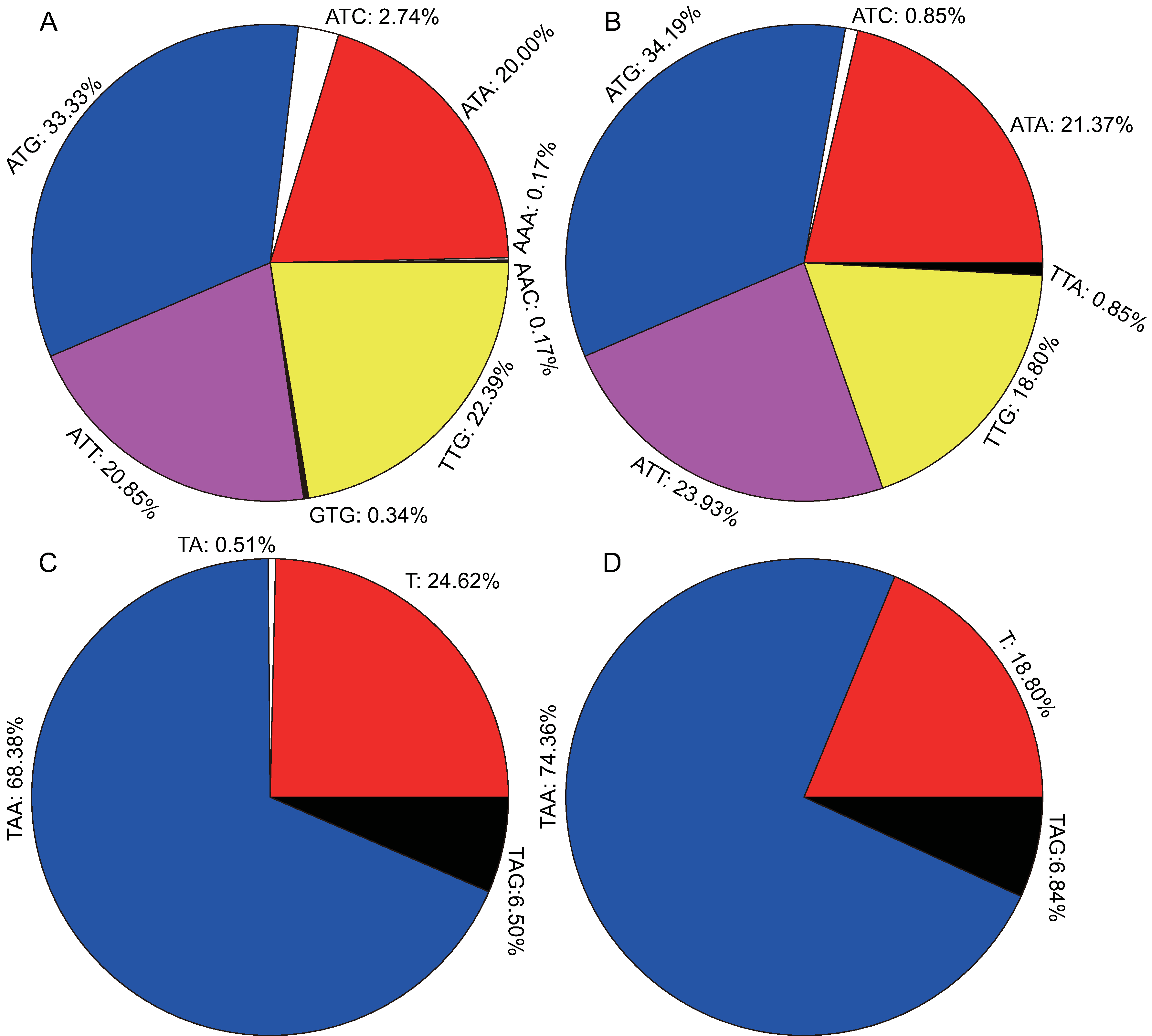

Supplement: S3 Fig — (A) Use of initiation codons in phytophagous bugs. (B) Use of initiation codons in predatory bugs. (C) Stop codon usage in phytophagous bugs. (D) Stop codon usage in predatory bugs. (TIF) [file pone.0292738.s003.tif]

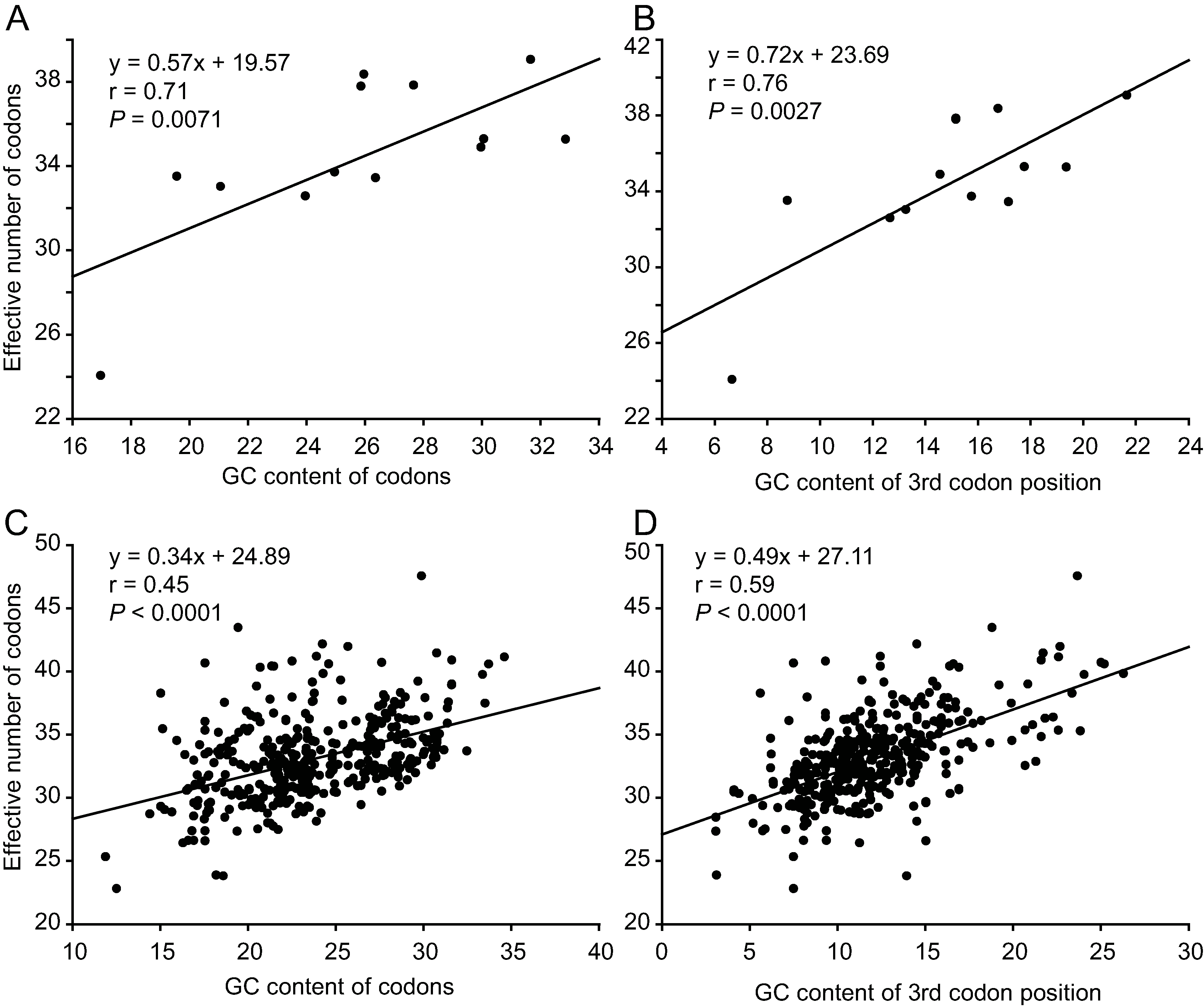

Supplement: S4 Fig — (A) Total G+C content of codons in Aelia fieberi. (B) G+C content of the third position of the codon in A. fieberi. (C) Total G+C content of codons in 52 species of Pentatomidae. (D) G+C content of the third position of the codon in 52 species of Pentatomidae. (TIF) [file pone.0292738.s004.tif]
