# Supplementary material for: Mitogenome of the stink bug Aelia fieberi (Hemiptera: Pentatomidae) and a comparative genomic analysis between phytophagous and predatory members of Pentatomidae
Source: PLoS One. 2023 Oct 11;18(10):e0292738. doi: 10.1371/journal.pone.0292738 (PMC10566676; doi:10.1371/journal.pone.0292738)
Supplement: S1 Table — (DOCX) [file pone.0292738.s005.docx]

**Table S1.** Annotation of the *Aelia fieberi* mitochondrial genome

| Gene | Strand | Position | Length | Intergenic nucleotide | Initiation codon | Stop codon | Anticodon |
| --- | --- | --- | --- | --- | --- | --- | --- |
| trnI | + | 1-66 | 66 | 8 |  |  | GAT |
| trnQ | - | 75-143 | 69 | 6 |  |  | TTG |
| trnM | + | 150-215 | 66 | 0 |  |  | CAT |
| **nad2** | + | 216-1202 | 987 | 18 | ATC | TAA |  |
| trnW | + | 1221-1286 | 66 | -8 |  |  | TCA |
| trnC | - | 1279-1344 | 66 | 3 |  |  | GCA |
| trnY | - | 1348-1412 | 65 | 6 |  |  | GTA |
| **cox1** | + | 1419-2958 | 1540 | 0 | ATA | T |  |
| trnL2 | + | 2959-3024 | 66 | 0 |  |  | TAA |
| **cox2** | + | 3025-3703 | 679 | 0 | ATA | T |  |
| trnK | + | 3704-3774 | 71 | 11 |  |  | CTT |
| trnD | + | 3786-3857 | 72 | 9 |  |  | GTC |
| **atp8** | + | 3867-4016 | 150 | -7 | ATA | TAA |  |
| **atp6** | + | 4010-4684 | 675 | 2 | ATG | TAA |  |
| **cox3** | + | 4687-5475 | 789 | 4 | ATG | TAA |  |
| trnG | + | 5480-5542 | 63 | 0 |  |  | TCC |
| **nad3** | + | 5543-5894 | 352 | 0 | ATA | T |  |
| trnA | + | 5895-5960 | 66 | 8 |  |  | TGC |
| trnR | + | 5969-6031 | 63 | 2 |  |  | TCG |
| trnN | + | 6034-6100 | 67 | -1 |  |  | GTT |
| trnS1 | + | 6100-6168 | 69 | 1 |  |  | GCT |
| trnE | + | 6170-6238 | 69 | -2 |  |  | TTC |
| trnF | - | 6237-6303 | 67 | 3 |  |  | GAA |
| **nad5** | - | 6307-8008 | 1702 | 1 | ATT | T |  |
| trnH | - | 8010-8073 | 64 | 3 |  |  | GTG |
| **nad4** | - | 8077-9405 | 1329 | -7 | ATG | TAA |  |
| **nad4l** | - | 9399-9686 | 288 | 2 | ATT | TAA |  |
| trnT | + | 9689-9753 | 65 | 0 |  |  | TGT |
| trnP | - | 9754-9819 | 66 | 7 |  |  | TGG |
| **nad6** | + | 9827-10309 | 483 | 4 | ATA | TAA |  |
| **cob** | + | 10314-11453 | 1140 | 8 | ATG | TAA |  |
| trnS2 | + | 11462-11530 | 69 | 24 |  |  | TGA |
| **nad1** | - | 11555-12478 | 924 | 0 | TTG | TAG |  |
| trnL1 | - | 12479-12543 | 65 | 0 |  |  | TAG |
| rrnL | - | 12544-13815 | 1272 | 0 |  |  |  |
| trnV | - | 13816-13883 | 68 | 0 |  |  | TAC |
| rrnS | - | 13884-14668 | 785 | 0 |  |  |  |
| Control region |  | 14669-15471 | 803 | 0 |  |  |  |
