# Supplementary material for: Mitogenome of the stink bug Aelia fieberi (Hemiptera: Pentatomidae) and a comparative genomic analysis between phytophagous and predatory members of Pentatomidae
Source: PLoS One. 2023 Oct 11;18(10):e0292738. doi: 10.1371/journal.pone.0292738 (PMC10566676; doi:10.1371/journal.pone.0292738)
Supplement: S2 Table — (DOCX) [file pone.0292738.s006.docx]

**Table S2.** Nucleotide composition of *Aelia fieberi* (%)

| Region | A% | T% | C% | G% | A+T% | G+C% | AT skew | GC skew |
| --- | --- | --- | --- | --- | --- | --- | --- | --- |
| atp6 | 37.04 | 35.26 | 16.89 | 10.81 | 72.30 | 27.70 | 0.02 | -0.22 |
| atp8 | 43.33 | 38.67 | 10.67 | 7.33 | 82.00 | 18.00 | 0.06 | -0.19 |
| cox1 | 34.16 | 32.92 | 17.73 | 15.19 | 67.08 | 32.92 | 0.02 | -0.08 |
| cox2 | 39.76 | 30.19 | 16.79 | 13.25 | 69.95 | 30.04 | 0.14 | -0.12 |
| cox3 | 34.47 | 33.84 | 17.24 | 14.45 | 68.31 | 31.69 | 0.01 | -0.09 |
| cob | 34.65 | 35.26 | 17.81 | 12.28 | 69.91 | 30.09 | -0.01 | -0.18 |
| nad1 | 24.57 | 50.22 | 8.55 | 16.67 | 74.79 | 25.22 | -0.34 | 0.32 |
| nad2 | 44.07 | 34.55 | 11.85 | 9.52 | 78.62 | 21.37 | 0.12 | -0.11 |
| nad3 | 39.77 | 34.09 | 15.63 | 10.51 | 73.86 | 26.14 | 0.08 | -0.20 |
| nad4 | 25.36 | 50.56 | 10.31 | 13.77 | 75.92 | 24.08 | -0.33 | 0.14 |
| nad4l | 24.65 | 49.65 | 11.46 | 14.24 | 74.30 | 25.70 | -0.34 | 0.11 |
| nad5 | 24.62 | 48.88 | 11.1 | 15.39 | 73.50 | 26.49 | -0.33 | 0.16 |
| nad6 | 39.75 | 40.79 | 11.39 | 8.07 | 80.54 | 19.46 | -0.01 | -0.17 |
| rrnL | 32.94 | 44.42 | 7.94 | 14.70 | 77.36 | 22.64 | -0.15 | 0.30 |
| rrnS | 33.89 | 42.17 | 8.79 | 15.16 | 76.06 | 23.95 | -0.10 | 0.27 |
| trnL2 | 35.38 | 38.46 | 9.23 | 16.92 | 73.84 | 26.15 | -0.04 | 0.29 |
| trnS2 | 40.58 | 37.68 | 10.14 | 11.59 | 78.26 | 21.73 | 0.04 | 0.07 |
| trnA | 34.85 | 34.85 | 15.15 | 15.15 | 69.70 | 30.30 | 0.00 | 0.00 |
| trnC | 34.85 | 34.85 | 13.64 | 16.67 | 69.70 | 30.31 | 0.00 | 0.10 |
| trnD | 44.44 | 37.5 | 9.72 | 8.33 | 81.94 | 18.05 | 0.08 | -0.08 |
| trnE | 43.48 | 40.58 | 8.7 | 7.25 | 84.06 | 15.95 | 0.03 | -0.09 |
| trnF | 35.82 | 37.31 | 11.94 | 14.93 | 73.13 | 26.87 | -0.02 | 0.11 |
| trnG | 42.86 | 36.51 | 11.11 | 9.52 | 79.37 | 20.63 | 0.08 | -0.08 |
| trnH | 31.25 | 40.63 | 6.25 | 21.88 | 71.88 | 28.13 | -0.13 | 0.56 |
| trnI | 40.91 | 33.33 | 10.61 | 15.15 | 74.24 | 25.76 | 0.10 | 0.18 |
| trnK | 35.21 | 33.8 | 16.9 | 14.08 | 69.01 | 30.98 | 0.02 | -0.09 |
| trnL | 42.42 | 36.36 | 9.09 | 12.12 | 78.78 | 21.21 | 0.08 | 0.14 |
| trnM | 37.88 | 27.27 | 19.7 | 15.15 | 65.15 | 34.85 | 0.16 | -0.13 |
| trnN | 37.31 | 38.81 | 10.45 | 13.43 | 76.12 | 23.88 | -0.02 | 0.12 |
| trnP | 37.88 | 43.94 | 6.06 | 12.12 | 81.82 | 18.18 | -0.07 | 0.33 |
| trnQ | 27.54 | 37.68 | 11.59 | 23.19 | 65.22 | 34.78 | -0.15 | 0.33 |
| trnR | 41.27 | 33.33 | 14.29 | 11.11 | 74.60 | 25.40 | 0.11 | -0.13 |
| trnS | 37.68 | 31.88 | 14.49 | 15.94 | 69.56 | 30.43 | 0.08 | 0.05 |
| trnT | 44.62 | 36.92 | 7.69 | 10.77 | 81.54 | 18.46 | 0.09 | 0.17 |
| trnV | 35.29 | 41.18 | 11.76 | 11.76 | 76.47 | 23.52 | -0.08 | 0.00 |
| trnW | 45.45 | 37.88 | 10.61 | 6.06 | 83.33 | 16.67 | 0.09 | -0.27 |
| trnY | 24.62 | 43.08 | 10.77 | 21.54 | 67.70 | 32.31 | -0.27 | 0.33 |
| Control region | 35.99 | 35.74 | 17.68 | 10.59 | 71.73 | 28.27 | 0.00 | -0.25 |
| Whole genome | 41.89 | 31.70 | 15.44 | 10.97 | 73.59 | 26.41 | 0.14 | -0.17 |
