# Supplementary material for: Mitogenome of the stink bug Aelia fieberi (Hemiptera: Pentatomidae) and a comparative genomic analysis between phytophagous and predatory members of Pentatomidae
Source: PLoS One. 2023 Oct 11;18(10):e0292738. doi: 10.1371/journal.pone.0292738 (PMC10566676; doi:10.1371/journal.pone.0292738)
Supplement: S3 Table — (DOCX) [file pone.0292738.s007.docx]

**Table S3.** Codon usage in the mitochondrial genome of *Aelia fieberi*

| Codon | Count | RSCU | Codon | Count | RSCU | Codon | Count | RSCU | Codon | Count | RSCU |
| --- | --- | --- | --- | --- | --- | --- | --- | --- | --- | --- | --- |
| UUU(F) | 241 | 1.56 | UCU(S) | 97 | 2.20 | UAU(Y) | 148 | 1.68 | UGU(C) | 41 | 1.74 |
| UUC(F) | 68 | 0.44 | UCC(S) | 17 | 0.39 | UAC(Y) | 28 | 0.32 | UGC(C) | 6 | 0.26 |
| UUA(L) | 303 | 3.48 | UCA(S) | 100 | 2.27 | UAA(*) | 8 | 1.78 | UGA(W) | 82 | 1.67 |
| UUG(L) | 61 | 0.70 | UCG(S) | 5 | 0.11 | UAG(*) | 1 | 0.22 | UGG(W) | 16 | 0.33 |
| CUU(L) | 53 | 0.61 | CCU(P) | 65 | 1.97 | CAU(H) | 52 | 1.41 | CGU(R) | 18 | 1.31 |
| CUC(L) | 11 | 0.13 | CCC(P) | 27 | 0.82 | CAC(H) | 22 | 0.59 | CGC(R) | 1 | 0.07 |
| CUA(L) | 84 | 0.96 | CCA(P) | 36 | 1.09 | CAA(Q) | 45 | 1.61 | CGA(R) | 30 | 2.18 |
| CUG(L) | 11 | 0.13 | CCG(P) | 4 | 0.12 | CAG(Q) | 11 | 0.39 | CGG(R) | 6 | 0.44 |
| AUU(I) | 309 | 1.63 | ACU(T) | 70 | 1.58 | AAU(N) | 147 | 1.63 | AGU(S) | 27 | 0.61 |
| AUC(I) | 70 | 0.37 | ACC(T) | 25 | 0.56 | AAC(N) | 33 | 0.37 | AGC(S) | 6 | 0.14 |
| AUA(M) | 280 | 1.77 | ACA(T) | 80 | 1.81 | AAA(K) | 85 | 1.55 | AGA(S) | 101 | 2.29 |
| AUG(M) | 37 | 0.23 | ACG(T) | 2 | 0.05 | AAG(K) | 25 | 0.45 | AGG(S) | 0 | 0 |
| GUU(V) | 80 | 1.84 | GCU(A) | 54 | 1.51 | GAU(D) | 50 | 1.41 | GGU(G) | 66 | 1.26 |
| GUC(V) | 6 | 0.14 | GCC(A) | 24 | 0.67 | GAC(D) | 21 | 0.59 | GGC(G) | 9 | 0.17 |
| GUA(V) | 81 | 1.86 | GCA(A) | 58 | 1.62 | GAA(E) | 69 | 1.60 | GGA(G) | 98 | 1.88 |
| GUG(V) | 7 | 0.16 | GCG(A) | 7 | 0.20 | GAG(E) | 17 | 0.40 | GGG(G) | 36 | 0.69 |

Note：4 genes used T as terminal codon.
