# Supplementary material for: Mitogenome of the stink bug Aelia fieberi (Hemiptera: Pentatomidae) and a comparative genomic analysis between phytophagous and predatory members of Pentatomidae
Source: PLoS One. 2023 Oct 11;18(10):e0292738. doi: 10.1371/journal.pone.0292738 (PMC10566676; doi:10.1371/journal.pone.0292738)
Supplement: Attachment — Submitted filename: response to reviewers.docx [file pone.0292738.s008.docx]

**Rebuttal letter to the editors and reviewers (original comments from the editors and reviewers are in blue, and our responses are in black)**

Comment 1: Journal Requirements: When submitting your revision, we need you to address these additional requirements. 1. Please ensure that your manuscript meets PLOS ONE's style requirements, including those for file naming. The PLOS ONE style templates can be found at <https://journals.plos.org/plosone/s/file?id=wjVg/PLOSOne_formatting_sample_main_body.pdf> and <https://journals.plos.org/plosone/s/file?id=ba62/PLOSOne_formatting_sample_title_authors_affiliations.pdf>

Response: Thank you for the comment! Our manuscript meets PLOS ONE's style requirements.

Comment 2: We note that the grant information you provided in the ‘Funding Information’ and ‘Financial Disclosure’ sections do not match. When you resubmit, please ensure that you provide the correct grant numbers for the awards you received for your study in the ‘Funding Information’ section. Thank you for stating the following financial disclosure: "The funders had no role in study design, data collection and analysis, decision to publish, or preparation of the manuscript." At this time, please address the following queries:

a)        Please clarify the sources of funding (financial or material support) for your study. List the grants or organizations that supported your study, including funding received from your institution.

b)        State what role the funders took in the study. If the funders had no role in your study, please state: “The funders had no role in study design, data collection and analysis, decision to publish, or preparation of the manuscript.”

c)        If any authors received a salary from any of your funders, please state which authors and which funders.

d)        If you did not receive any funding for this study, please state: “The authors received no specific funding for this work.”

Please include your amended statements within your cover letter; we will change the online submission form on your behalf.

 Response: We provide the grant numbers as follows: This research was funded by National Natural Science Foundation of China, grant number 32060124; Guizhou Normal University, grant number Qianshixinmiao [2021]A11. The funders had no role in study design, data collection and analysis, decision to publish, or preparation of the manuscript.

Comment 3: Thank you for stating the following in your Competing Interests section: "the authors declare no conflicts of interest." Please complete your Competing Interests on the online submission form to state any Competing Interests. If you have no competing interests, please state "The authors have declared that no competing interests exist.", as detailed online in our guide for authors at <http://journals.plos.org/plosone/s/submit-now>. This information should be included in your cover letter; we will change the online submission form on your behalf.

 Response: We revised "the authors declare no conflicts of interest" as “The authors have declared that no competing interests exist”.

Comment 4: PLOS requires an ORCID iD for the corresponding author in Editorial Manager on papers submitted after December 6th, 2016. Please ensure that you have an ORCID iD and that it is validated in Editorial Manager. To do this, go to ‘Update my Information’ (in the upper left-hand corner of the main menu), and click on the Fetch/Validate link next to the ORCID field. This will take you to the ORCID site and allow you to create a new iD or authenticate a pre-existing iD in Editorial Manager. Please see the following video for instructions on linking an ORCID iD to your Editorial Manager account: <https://www.youtube.com/watch?v=_xcclfuvtxQ>

 Response: We added the ORCID iD of the corresponding author (0000-0003-3533-0731).

Comment 5: Additional Editor Comments: The study describes the whole mitogenome of the stink bug Aelia fieberi and provides comparative genomics and phylogeny of members of Pentatomidae. In general, the authors have used appropriate methods for mitogenome sequencing, assembly, annotation and comparative mitogenomic study along with phylogenetic analysis. However, there are some issues with the manuscript that authors will have to address before the manuscript can be considered further for review. Both major and minor issues are explained below in no particular order. (1) English grammar: One of the major problems with the manuscript is the English grammar, which needs thorough editing. Authors should take help of native English speaker or professional English editing services to overcome this issue. It is impossible to provide detailed comments on English grammar as almost every other line has a problem. But just to give an example, authors mention “The phylogenetic tree showed that Asopinae and Phyllocephalinae were monophyly, however, Pentatominae and Podopinae were not monophyly.” In both the cases in the above sentence authors should have used “monophyletic” rather than “monophyly”.

 Response: Thank you for the comment. English grammar has been checked by a native English speaker.

Comment 6: Mitogenome GenBank accession numbers: NCBI GenBank numbers for mitogenomes that start with "NC_" are "Provisional REFSEQ" numbers and should be replaced by the corresponding finally GenBank accession number after NCBI review. For example, the provisional refseq number for the sequence published in this manuscript is NC_067750 and the final GanBank number is OL631608. Authors should use the final GenBank numbers, if available, in all cases throughout the manuscript.

 Response: Thank you for the comment! All provisional GenBank numbers were replaced with final GenBank numbers.

Comment 7: Comparative genomic analysis: The strong point of the manuscript is the comparative genomic analysis between the phytophagous and predatory members of Pentatomoidae and not the phylogenetic analysis. This is because the paraphyly of subfamilies in the family Pentatomoidae is already well known and the results obtained in the present study are similar to earlier reports. So, in the title, rather than talking about the implications of the study with respect to phylogeny, authors should focus more on the comparative genomics of phytophagous and predatory members of Pentatomoidae. Accordingly, authors should revise the introduction by providing more information about the phytophagous and predatory members of Pentatomoidae. Further, authors should increase the taxonomic sampling for predatory members of Pentatomoidae based on mitogenome information available on NCBI GenBank database and redo the analysis. In the phylogenetic tree, authors can highlight the phytophagous and predatory members of Pentatomoidae separately.

 Response: Thank you for the comment! We added more information about phytophagous and predatory bugs. Owing to very few species of Pentatomidae are predatory stink bugs, all mitogenomes of predatory stink bugs available in GenBank were used for analysis. Predatory bugs and phytophagous bugs have been highlighted in pink and yellow background, respectively.

Comment 8: Manuscript preparation: In the results authors should first describe the mitogenome of Aelia fieberi, including information on protein coding genes, codon usage, tRNA, etc. (make sure that current fig 6 is after fig 1) and then provide phylogeny and comparative genomics between phytophagous and predatory members of Pentatomoidae in separate subheadings.

 Response: Thank you for the comment! Firstly, we described the mitogenome; then showed the results of phylogenetic tree, lastly, described the results of comparative genomics between phytophagous and predatory stink bugs. Correspondingly, subheadings were listed at the beginning of each part.

Comment 9: Phylogenetic analysis: Provide maximum likelihood analysis along with Bayesian analysis.

 Response: Maximum likelihood tree was added (Fig S2).

Comment 10: Focus on phylogeny: As stated earlier, authors should remove the focus on phylogenetic relationships among Pentatomoidae as it is not the strong point of the manuscript. Statements such as, "The mitogenomic information of A. fieberi could shed light on the evolution of Pentatomoidae" make little sense because the resulrts shown by authors are same as what earlier studies have shown and the additional sequence of Aelia fieberi mitogenome does not provide any new insights on the issue. What authors should focus on is the comparative genomices. The importance of the mitogenome of Aelia fieberi can be explained by suggesting filling the knowledge gap for this important crop pest.

 Response: Thank you for the comment! We described as follows: the mitogenomic information of *A. fieberi* could fill the knowledge gap for this important crop pest. The differences between phytophagous bugs and predatory bugs deepen our understanding of the effect of feeding habit on mitogenome.

Comment 11: Please use the space provided to explain your answers to the questions above. You may also include additional comments for the author, including concerns about dual publication, research ethics, or publication ethics. (Please upload your review as an attachment if it exceeds 20,000 characters).

 Response: We answered these questions point by point. The manuscript has not been published in whole or in part nor is it being considered for publication elsewhere.

Comment 12: Reviewer #1: This paper reports the mitochondrial genome data of Aelia fieberi, which can provide some data support for the phylogeny of Pentatomidae or Pentatomoidea. However, the content of the paper is relatively simple, and the analysis method is not innovative, the analysis of the results is also innovative.

 Response: Thank you for the comment! The highlight of the manuscript is the comparative analysis of mitogenomes of phytophagous and predatory bugs.

Comment 13: Reviewer #2: Review of manuscript Mitogenome of the stink bug Aelia fieberi (Hemiptera: Pentatomidae) and a phylogenetic analysis of Pentatomidae (PONE-D-23-11789). In this study, authors sequenced and analyzed the complete mitogenome of Aelia fieberi. Similarly, authors compared the mitogenome of Aelia fieberi with 53 available complete mitogenomes of bugs of the Pentatomidae family. The analysis revealed that phytophagous bugs and predatory bugs had different evolutionary rates. Analysis also revealed that Asopinae and Phyllocephalinae are monophyly and Pentatominae and Podopinae are not monophyly groups. Authors suggested that the phylogenetic relationships of Pentatomoidae are complex, and need revaluation and revision. The current study represents the comparative mitogenomic and phylogenetic analyses within the Pentatomoidae family and provides better insights into the mitogenome features and evolution of bugs belonging to the Pentatomoidae family.

The overall study is original, relevant, and interesting but requires improvements in data analysis, results, and discussion sections of the manuscript. The statistical analysis and interpretations based on genetic analysis are appropriate. However, in-depth analysis, such as usage bias of start and stop codons, non-synonymous/synonymous ratios (ω = dN/dS ) in protein-coding genes of mitogenomes, gene rearrangement, and the divergence time analysis of Pentatomidae bugs will strengthen the results and discussion section of the manuscript. Generally, a strong codon usage bias is reported in Hemiptera. Authors should comment on an effective number of codon (ENC) values for Aelia fieberi and compare them with the other hemipteran species. Standard ENC curve (ENC vs G/C composition of the 3rd position of codons), will provide better information about the codon usage patterns in Hemipteran species.

Response: Thank you for the comment! Gene rearrangement of species of Pentatomidae was added in Fig S1. The results of the start and stop codons were added in Fig S3. The results of non-synonymous/synonymous ratios (ω = dN/dS) in protein-coding genes of mitogenomes were presented in Fig 7. Owing to lack of data of fossil, the divergence time analysis of stink bugs was not performed in our study. The effective number of codons had a liner relationship with the total GC content of the codons (Fig S4A) and GC content of the third position of the codons (Fig S4B) in *A.* *fieberi*. A similar relationship occurred in other species of Pentatomidae with a complete mitogenome sequence (Fig S4C, D).

With the above modifications, we think that the revised manuscript has been substantially improved in terms of data presentation and analyses, and appreciate your consideration for its publication in PLOS ONE.

Best regards,

Yeying Wang, Ph.D.

School of Life Sciences

Guizhou Normal University

E-mail: [yeyingwang@gznu.edu.cn](mailto:yeyingwang@gznu.edu.cn)
